# A Model of Stimulus-Specific Neural Assemblies in the Insect Antennal Lobe

**DOI:** 10.1371/journal.pcbi.1000139

**Published:** 2008-08-01

**Authors:** Dominique Martinez, Noelia Montejo

**Affiliations:** 1 LORIA, Campus Scientifique, Vandoeuvre-lès-Nancy, France; 2 INRA, UMR 1272 Physiologie de l'insecte, Versailles, France; University College London, United Kingdom

## Abstract

It has been proposed that synchronized neural assemblies in the antennal lobe of insects encode the identity of olfactory stimuli. In response to an odor, some projection neurons exhibit synchronous firing, phase-locked to the oscillations of the field potential, whereas others do not. Experimental data indicate that neural synchronization and field oscillations are induced by fast GABA_A_-type inhibition, but it remains unclear how desynchronization occurs. We hypothesize that slow inhibition plays a key role in desynchronizing projection neurons. Because synaptic noise is believed to be the dominant factor that limits neuronal reliability, we consider a computational model of the antennal lobe in which a population of oscillatory neurons interact through unreliable GABA_A_ and GABA_B_ inhibitory synapses. From theoretical analysis and extensive computer simulations, we show that transmission failures at slow GABA_B_ synapses make the neural response unpredictable. Depending on the balance between GABA_A_ and GABA_B_ inputs, particular neurons may either synchronize or desynchronize. These findings suggest a wiring scheme that triggers stimulus-specific synchronized assemblies. Inhibitory connections are set by Hebbian learning and selectively activated by stimulus patterns to form a spiking associative memory whose storage capacity is comparable to that of classical binary-coded models. We conclude that fast inhibition acts in concert with slow inhibition to reformat the glomerular input into odor-specific synchronized neural assemblies.

## Author Summary

A fundamental question in computational neuroscience is to understand how interactions between neurons underlie sensory coding and information storage. In the first relay of the insect olfactory system, odorant stimuli trigger synchronized activities in neuron populations. Synchronized assemblies may arise as a consequence of inhibitory coupling, because they are disrupted when inhibition is pharmacologically blocked. Using computational modelling, we studied the role of inhibitory, noisy interactions in producing stimulus-specific synchrony. So far, experimental data and modelling studies indicate that fast inhibition induces neural synchrony, but it remains unclear how desynchronization occurs. From theoretical analysis and computer simulations, we found that slow inhibition plays a key role in desynchronizing neurons. Depending on the balance between fast and slow inhibitory inputs, particular neurons may either synchronize or desynchronize. The complementary roles of the two synaptic time scales in the formation of neural assemblies suggest a wiring scheme that produces stimulus-specific inhibitory interactions and endows inhibitory sub-circuits with properties of binary memories.

## Introduction

The primary olfactory center of insects, the antennal lobe (AL), is a network of excitatory projection neurons (PNs) interconnected via inhibitory local neurons (LNs). Such excitatory-inhibitory architectures are known to produce network oscillations [1],[2],[3]. Field potential oscillations have been observed in the AL of the locust [4],[5], of the bee [6] and the moth [7],[8]. The oscillations persist after ablation of higher brain structures involved in olfaction and are thus attributable to the AL and, in particular, to the synchronization of the underlying PNs. It has been proposed that odors are encoded by distinctive synchronized neural assemblies [4],[5],[9]. These assemblies do not only encode sensory information but also store short-term memories [10]. At the same time as synchronized assemblies are formed, other neurons have to desynchronize in order to avoid pathological epileptic-like hypersynchronization. In the olfactory system, it turns out that desynchronization might also be important for neural processing, as synchronized and desynchronized neurons carry qualitatively different information about the odorant [11]. What then are the synaptic mechanisms responsible for synchronization and desynchronization?

It is now clear that PN synchrony results from the interplay with GABAergic LNs, and more specifically from ionotropic GABA_A_ receptors. Neural synchronization and field potential oscillations are lost when GABA_A_ inhibition is pharmacologically blocked by local injection of picrotoxin into the AL of the locust [12], of the honeybee [10] and the moth [9]. Picrotoxin desynchronizes neural assemblies and impairs discrimination of similar odors in the honeybee [10],[13]. However, picrotoxin does not affect the slow phases of inhibition observed in PNs [12],[14] and, thus, multiple inhibitory pathways are likely to be present in the insect AL. In the honeybee, a second inhibitory network has been shown to be picrotoxin-insensitive and glomerulus-specific [15], and histamine has been proposed as the second inhibitory transmitter [16]. Experimental studies as well as computational modelling postulated the existence of slow inhibition [12],[17]. The presence of a second inhibitory network mediated by metabotropic GABA_B_ receptors has been shown in the *Drosophila* AL [14]. GABA_B_ postsynaptic potentials present a much slower decay rate than the ones produced by GABA_A_ inhibition. Interestingly, little evidence for oscillation and synchronization has been found in *Drosophila* [14] (but see [18]). In addition, spike timing precision increases in PNs when GABA_B_ inhibition is pharmacologically blocked [14].

These observations suggest a synchronizing and desynchronizing effect of fast and slow inhibition, respectively. Without a better understanding of the role of GABAergic synapses, however, it is difficult to evaluate what such synchronization reveals about olfactory coding. Here, using computational modelling, we test the hypothesis that fast GABA_A_-type inhibition synchronizes whereas slow GABA_B_-type inhibition desynchronizes. Previous theoretical studies have shown that inhibitory networks synchronize (e.g. [19],[20]) and that cell heterogeneity or noise added to the input affects the synchronization properties (e.g. [21],[22]). Although synaptic transmission can be very unreliable in biological neural networks, none of the modelling studies has explored the effect of synaptic failures. The probability of synaptic failure has been shown to be 0.7 in hippocampal pyramidal neurons [23] and ∼0.5 for dendrodendritic synapses between Mitral and Granule Cells in the olfactory bulb [24]. Is there any computational advantage for this synaptic unreliability? Does it affect spike timing precision and neural synchrony? As we show both theoretically and by computer simulations, failures in synaptic transmission are especially tolerated with fast GABA_A_ synapses but not with slow GABA_B_ synapses. We also demonstrate that the relative amount of received fast and slow inhibition regulates synchrony and determines whether particular neurons engage in neural assemblies. Finally, the complementary roles of GABA_A_ and GABA_B_ synapses in the formation of neural assemblies suggest a wiring scheme that produces stimulus-specific spatial patterns of inhibition in the antennal lobe.

Throughout the paper, we use computational models of increasing complexity. We first use a model of uncoupled PNs to determine whether the injection of a hyperpolarizing current step enhances spike timing precision. We then use a model of PNs coupled with GABA_A_ and GABA_B_ unreliable synapses to understand the effect of synaptic failures on neural synchrony. We finally propose a stimulus-dependent gating mechanism of lateral inhibition between PNs and use Hebbian learning to store and recall stimulus patterns in inhibitory sub-circuits.

## Results

### Enhancement of Spike Timing Precision with Somatic Injection of Hyperpolarizing Current

**Figure 1:**
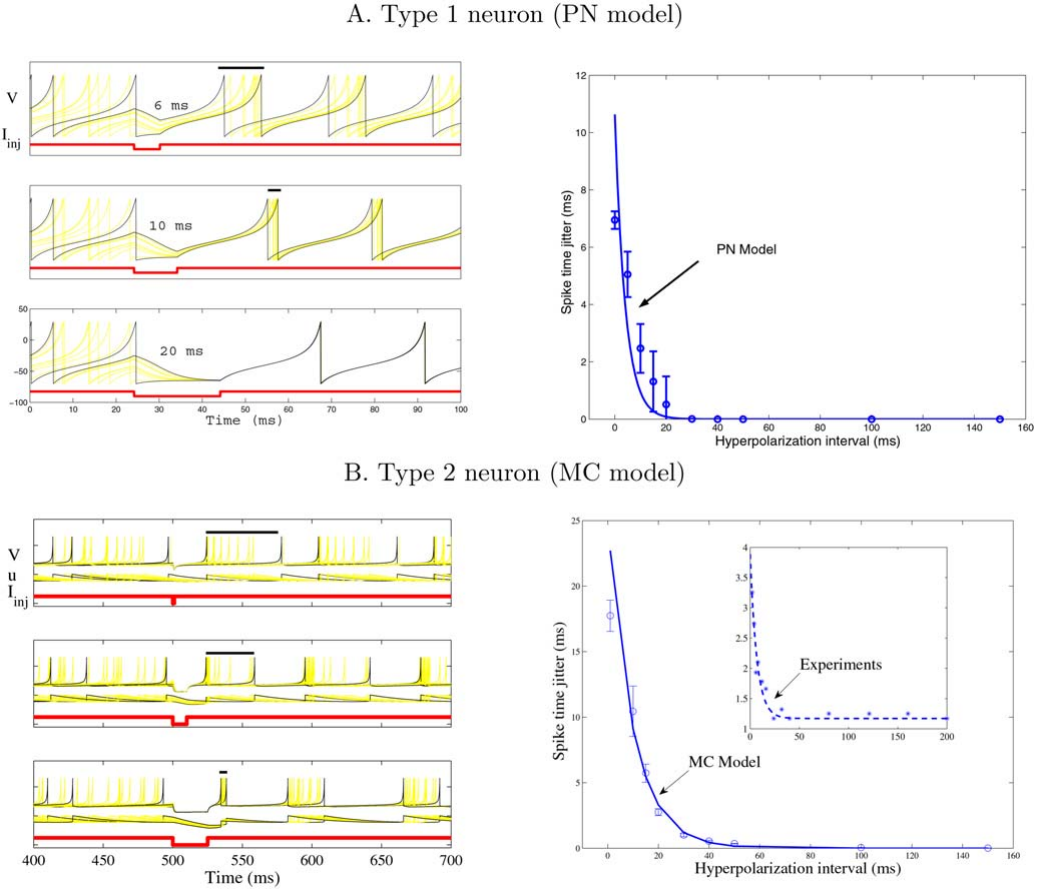
Spike timing precision with somatic injection of hyperpolarizing current. (A) is for our type 1 model of Projection Neuron (see Methods). Left: temporal evolution of the membrane potential *V* with somatic injection of hyperpolarizing current pulses *I*_inj_ of different durations (6, 10, and 20 ms). The spike time jitter (bars above the spikes) is estimated as the temporal dispersion of the first spikes right after inhibition. Right: spike time jitter versus duration of the hyperpolarizing interval. Means and standard deviations are estimated over five runs; The solid curve is an exponential fit of the data (time constant = 4.1 ms). (B) is for a type 2 model of olfactory bulb Mitral Cell. Left: temporal evolution of the state variables (membrane potential *V* and adaptive current *u*) for different durations of the hyperpolarizing current (1, 10, and 25 ms). Right: spike time jitter versus duration of the hyperpolarizing interval. Same convention as in (A) (time constant of exponential fit = 9.8 ms). Figure inset represents the exponential fit of experimental data recorded in MCs in vitro (time constant = 6.8 ms), modified from [28], Figure 4A4.

First, we consider a population of uncoupled PNs modelled as integrate-and-fire neurons with nonlinear spike generating current (Equation 10 with *I*_syn_ = 0, see Methods). Their initial membrane potential is chosen randomly so that the PN population is completely desynchronized. To check whether inhibition synchronizes, we mimic inhibitory current injection into PNs and vary the duration of the hyperpolarizing pulse. Figure 1A left shows representative voltage traces for hyperpolarization intervals of 6, 10, and 20 ms. Hyperpolarized PNs have a tendency to relax to their resting potential *V*_rest_, given by Equation 11 (see Methods), and forget their initial states so that they fire synchronously when inhibition stops. The spike time jitter was calculated as the temporal dispersion of the first spikes right after inhibition. It is well fitted with a single exponential (4.1 ms time constant, Figure 1A right). Enhancement of spike timing precision is attributable to a loss of initial conditions and can be interpreted in terms of *transient resetting*, as theoretically described in [25]. In the case of our PN model, the injected hyperpolarizing current pulse allows the integrate-and-fire neuron to jump from a repetitive spiking regime to a steady state (resting potential) across a saddle node bifurcation characteristics of type 1 excitability [26].

To check whether transient resetting is also effective for other types of neurons, we repeated the simulations with a model of olfactory mitral cells (MCs) that displays type 2 excitability [27]. This MC model has two variables (membrane potential and adaptive current) which relax to their fixed point during the phase of inhibition (see Figure 1B, left). Thus, injection of hyperpolarizing current plays a similar role in type 1 and type 2 neurons. Precise spike timing is obtained for hyperpolarization intervals of longer duration because there is enough time for variables, such as membrane potential or adaptive current, to reach their steady state and forget their initial conditions. The decay rate of the spike time jitter for the MC model is well fitted with a single exponential (time constant = 9.8 ms, Figure 1B, right). It is also in line with the one estimated from experimental data recorded in MCs in vitro [28] (time constant = 6.8 ms, inset in Figure 1B, right).

Altogether these observations suggest that inhibition may play a role in enhancing spike timing precision in PNs, since it tends to eliminate the influence of initial conditions. Because long-lasting inhibition leaves more time for transient resetting, one can speculate that precise spike timing would be achieved with GABA_B_-type inhibition. Evidence in favour of this hypothesis is provided by in vitro recordings in MCs [28] for which smaller spike time jitter is obtained with somatic current injection of longer duration (Figure 1B, inset). Therefore, one would expect higher spike time jitter in vivo when slow GABA_B_ inhibition is pharmacologically blocked. Application of a GABA_B_ antagonist in the *Drosophila* AL, however, shows just the opposite (see Figure 4 in [14]). To understand this paradox, we simulate neuron models coupled with GABA_A_ or GABA_B_ synapses in the next section.

### Impact of Synaptic Unreliability on Spike Timing Precision

**Figure 2:**
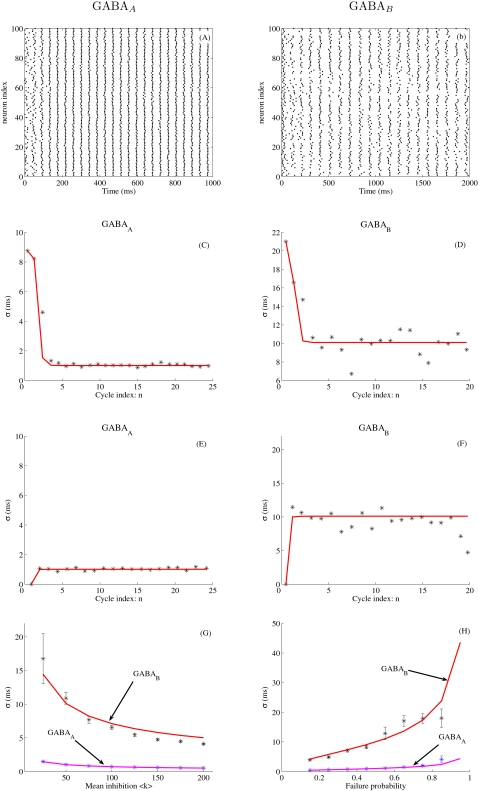
Spike timing precision with GABA_A_ or GABA_B_ inhibition. In (A–G), the failure probability is *P*_failure_ = 0.5. (A): Spike rasterplot for GABA_A_ coupling. The peak GABA_A_ conductance is *g_a_* = 1 nS. The frequency of the network oscillation is F ∼20 Hz. (B) Spike rasterplot for GABA_B_ coupling (*g_b_* = 0.1 nS, F ∼10 Hz). (C,D) Temporal evolution of the spike time jitter *σ*(*n*), where *n* is the index of the oscillatory cycle. Convergence is reached in about 3 cycles, i.e., 300 ms with GABA_B_ and 150 ms with GABA_A_. The initial condition is the desynchronized state (see Methods). (E,F) Same conventions as in (C–D), except that the initial condition is now the synchronized state. (G) Spike time jitter *σ* obtained at convergence (*σ*(*n*) averaged over the last two oscillatory cycles) as a function of the mean inhibitory drive 〈*k*〉 received by the neurons (the number of neurons *N* scales from 50 to 400). (H) *σ* as a function of the failure probability *P*_failure_. In (C–H), the stars represent the spike time jitter estimated from simulations (see Methods, means and standard deviations estimated over 10 runs). The solid curves are for theoretical values obtained from Equation 1 (in C–F) or from Equation 2 (in G–H).

We consider two distinct networks of *N* = 100 neurons completely connected, one with fast GABA_A_ synapses (*τ*_GABA_ = 10 ms) and another with slow GABA_B_ synapses (*τ*_GABA_ = 100 ms). Since chemical synapses are believed to be quite unreliable [23], a probability of synaptic failure *P*_failure_ is taken into account. Rasterplots in Figure 2A and 2B present network oscillations in the presence of fast or slow inhibition, the frequency being higher with fast inhibition (F ∼20 Hz with GABA_A_ and F ∼10 Hz with GABA_B_). As revealed by Equation A-1 (see Text S1), the period *T* of the network oscillations grows as ln *g* where *g* is the peak synaptic conductance. The period is thus quite robust to changes in the strength of inhibition. However, it depends linearly on the decay time *τ*_GABA_ of the inhibitory synapse. This observation is in agreement with simulation results (see Figure S1) and with previous studies, e.g., [29]. In Figure 2A and 2B, the PN population is partially synchronized but with higher temporal dispersion in the presence of slow inhibition. We now quantify analytically the temporal dispersion of the spiking events within each cycle. As shown in Text S1 (Equation A-3), the spike time jitter *σ* ^2^(*n*) of the PN population at the *n*-th cycle can be expressed as a simple linear recursive relation
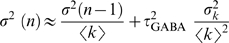
(1)where 〈*k*〉 = *N*(1−*P*_failure_) and *σ_k_* ^2^+*NP*_failure_ (1−*P*_failure_) are the mean and variance in the number *k* of inhibitory synaptic events received by the PNs at each cycle. Note that the mathematical analysis in Text S1 did not take into account the PNs that do not receive any inhibition. Equation 1 is therefore not valid when *P*_failure_ = 1. Figure 2C and 2D compares the theoretical jitter *σ* ^2^(*n*) given by Equation 1 to the one obtained from simulations (see Methods). From the figure, we see that the spike time jitter reaches a stable state in about *n* = 3 cycles (300 ms with GABA_B_ versus 150 ms with GABA_A_). This stable state does not depend on initial conditions (compare Figure 2C and 2D with Figure 2E and 2F) but does depend on the time constant of the inhibitory synapse : *σ*≈1 ms for GABA_A_ and *σ*≈10 ms for GABA_B_. From Equation 1, the spike time jitter obtained at convergence is given by
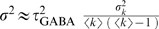
(2)


Figure 2G and 2H compares the theoretical *σ* to the one obtained from simulations. From Equation 2, *σ* is small when 〈*k*〉 is large, as confirmed in Figure 2G. Thus, variable inhibition is especially tolerated as the number of inhibitory inputs per cell is large. From Equation 2, loss of spike timing precision (high *σ* ^2^) is attributable to variance in the amount of received inhibition *σ_k_* ^2^. This variance comes from the presence of synaptic failures in our model (or from heterogeneous connectivity as we will see later). Because *σ* is proportional to the decay time constant of the inhibitory synapse, slow inhibition amplifies synaptic noise and leads to unpredictible firings. This finding can be noted in Figure 2H where *σ*>10 ms with slow GABA_B_ synapses for *P*_failure_≥0.5. In contrast, variable inhibition is especially tolerated with fast GABA_A_ synapses since *σ*<5 ms for any value of *P*_failure_. Equation 2 also holds for extended AL models taking into account lateral excitation between PNs (Figure S2) and considering inhibitory local neurons (Figure S3).

Our results predict that the loss of spike timing precision is attributable to variable inhibition received on slow GABA_B_-type synapses. Variable inhibition may come from hererogeneous connectivity or from the presence of synaptic failures, both of them being likely to occur in vivo. Thus, blocking GABA_B_ inhibition leads to enhanced spike timing precision (Figure 4 in [14]). In contrast, in vitro injection of hyperpolarizing current pulses, as done in [28], does not present such a variability. This explains the apparent contradiction between in vivo and in vitro experimental data, as noticed in the previous section.

### Asynchronous GABA Release Produces Long-Lasting Inhibition and Accentuates Temporal Dispersion

**Figure 3:**
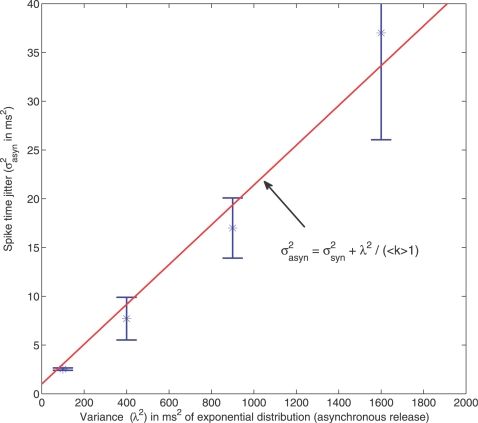
Spike timing precision with asynchronous GABA release. The stars represent the spike time jitter *σ* ^2^ estimated from simulations with asynchronous GABA release. For the simulations, we considered a network of *N* = 100 neurons coupled all-to-all with fast GABA_A_ synapses (*τ*_GABA_ = 10 ms). Each presynaptic spike triggers 10 post-synaptic events, released asynchronously according to an exponential distribution of variance *λ* ^2^ (Equation 14 in Methods). The solid line is given by Equation 4.

Inhibitory cells may release transmitters synchronously or asynchronously [30],[31]. In the olfactory bulb for example, GABAergic inhibition released by Granule Cells and received by Mitral Cells is asynchronous and variable across repeated trials [32],[33]. What might be the effect of asynchronous GABA release on the spike timing precision? As shown in Text S1 (Equation A-4), the spike time jitter *σ* ^2^(*n*) of the PN population at the *n*-th cycle is
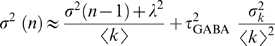
(3)where *λ* is the time constant of the exponential release distribution (Equation 14 in Methods). A high value of *λ* models the effect of asynchronous inhibition, where synaptic events may be released well after the arrival of an action potential on a synapse. On the contrary, a lower value of *λ* models the effect of synchronous inhibition. When *λ* = 0, Equation 3 becomes equivalent to Equation 1. At convergence of Equation 3, we have *σ* ^2^(*n*) = *σ* ^2^(*n*−1) = *σ*_asyn_^2^ and
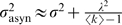
(4)where *σ* ^2^ is the spike time jitter obtained in the case of synchronous GABA release and is simply given by Equation 2. Asynchronous release accentuates temporal dispersion by adding the extra term *λ* ^2^/(〈*k*〉−1). Figure 3 compares the theoretical σ_asyn_^2^ to the one obtained from simulations for different values of *λ* ^2^. For the simulations, we considered a network of *N* = 100 neurons coupled all-to-all with fast GABA_A_ synapses (*τ*_GABA_ = 10 ms, *g_a_* = 1 nS, *P*_failure_ = 0.5). For *λ* = 0 ms (synchronous release), we have *σ*_asyn_ = *σ* = 1 ms (temporal dispersion obtained with GABA_A_ synapses, see previous section). We observe that *σ*_asyn_^2^ increases linearly with *λ* ^2^, as predicted by Equation 4. From Equation 4, *σ*_asyn_ = 10 ms when *λ* = 70 ms, which is the same level of temporal dispersion as the one obtained with synchronous release and slow GABA_B_ synapses (*τ*_GABA_ = 100 ms, see previous section). The loss of spike-timing precision is thus achieved with asynchronous release, despite fast GABA_A_ synapses. Actually, the asynchronous synaptic events sum gradually over time so as to produce a resulting inhibition which decays with a time constant approximately equal to *λ* (when *λ* is large as shown previously [34]). Asynchronous release can be seen as a way to produce long-lasting inhibition despite the fast decay time of individual events mediated by GABA_A_ receptors.

### GABA_A_ and GABA_B_ Synapses Play Opposite Roles in Synchronization

**Figure 4:**
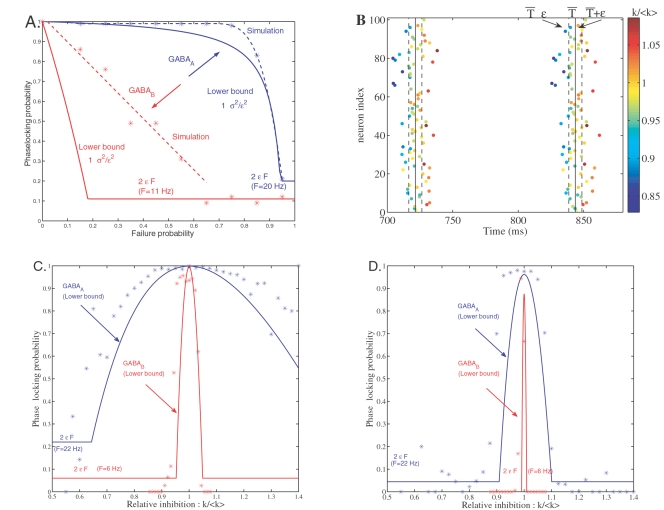
Phase-locking probability with GABA_A_ or GABA_B_ inhibition. (A) Phase-locking probability versus probability of synaptic failure in homogeneous networks. The stars represent data estimated from the simulations in the presence of GABA_A_ (blue stars) or GABA_B_ (red stars). The resolution at which phase-locked spikes are determined is *ε* = 5 ms (B). The solid curves are for the lower bounds on the phase-locking probability (Equation 5). The constant value 2*εF* (horizontal lines) is for the desynchronized state corresponding to the case where the firings are uniformly distributed over the duration 1/*F* of the oscillatory cycle. (B) Spike rasterplot over two consecutive oscillatory cyles. Synchronized spikes are those which fall within a temporal bin of ±*ε* around the mean firing time *T̅* of the PN population. Dots with the same color correspond to the spikes fired by the neurons receiving the same amount of inhibition (*k*/〈*k*〉). The number of inhibitory inputs received by a particular cell is *k* and the inhibition received on average by the neuronal population is 〈*k*〉. Synchronized neurons are those for which *k*≈〈*k*〉. (C) Phase-locking probability versus relative amount of received inhibition (*k*/〈*k*〉) in heterogeneous networks (probability of connection = 0.4 with GABA_A_ and 0.9 with GABA_B_). The resolution at which phase-locked spikes are determined is *ε* = 5 ms. The lower bounds on the phase-locking probability are given by Equation 6. (D) Same conventions as in (C), except that *ε* = 1 ms.

A classical approach for measuring synchrony is to consider that a spike occuring at time *T* is phase-locked when *T* is within a temporal window of ±*ε* ms around the mean firing time *T̅* of the neuronal population. The relative count of these synchronous events among the population of neurons provides an estimate of the phase-locking probability. A theoretical lower bound is given by direct application of the Bienaymé-Tchebyshev inequality
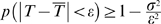
(5)where *σ* ^2^ depends on *P*_failure_ via Equation 2. Figure 4A compares the theoretical bound given by Equations 5 and 2 to the phase-locking probability estimated from simulations (*ε* = 5 ms, see Methods). The bound has the same, monotonically decreasing, behavior as the estimated probability. For both types of inhibition, the phase-locking probability decreases with *P*_failure_ until it reaches a constant value (2*εF*, horizontal line in Figure 4A). This desynchronized state corresponds to the case where PN firings are uniformly distributed over the duration of the oscillatory cycle (1/*F*). With GABA_A_-type inhibition, the phase-locking probability decreases in a nonlinear way. More important is the presence of a plateau for *P*_failure_<0.7 which maintains a high probability of synchrony despite unreliable synapses. In contrast, the phase-locking probability decreases linearly with *P*_failure_ for GABA_B_-type inhibition. Thus, a small amount of synaptic noise on GABA_B_ synapses is sufficient to degrade synchronization in homogeneous networks.

In heterogeneous networks, the number of inhibitory inputs differs from one cell to another. Is the heterogeneity in connectivity sufficient to break synchrony in the absence of synaptic failure? As seen in Figure 4B, the number of inhibitory inputs has an influence on synchrony. The neurons which receive an amount *k* of inhibition very different than the mean inhibition 〈*k*〉 fire far away from the population and, thus, are not synchronized. Synchronized neurons are those for which *k*≈〈*k*〉. In the following, we analytically quantify the conditional probability that particular neurons receiving *k* inhibitory synaptic events fire in synchrony with the neuronal population. A lower bound on this conditional probability was previously derived in [35] (Equations 3.7 and 3.8) as
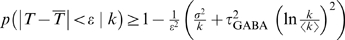
(6)


Here *σ* ^2^ is given by Equation 2. We have checked numerically that Equation 6 is a good candidate for the phase-locking probability. Figure 4C and 4D compares the lower bound given by Equations 6 and 2 to estimated data obtained from simulations. Both for GABA_A_ and GABA_B_ type inhibition, the phase-locking probability is an inverted U-function centered on the inhibition 〈*k*〉 received on average by the neurons. The existence of this inverted U-function does not depend on a specific choice for *ε* (*ε* = 5 ms in Figure 4C and 1 ms in Figure 4D). If a cell receives either a fairly large or a fairly small amount *k* of inhibition relative to the mean inhibitory drive 〈*k*〉, then it is likely that it will fire very far away from the other cells and thus will not be synchronized. A synchronization window is defined by the values of *k* for which the phase-locking probability is higher than a given threshold. With GABA_B_, the phase-locking probability becomes very sharp so that only neurons for which *k* = 〈*k*〉 are synchronized (very small synchronization window). Therefore, variable inhibition received on slow GABA_B_ synapses leads to desynchronization. In contrast, variable inhibition is especially tolerated with fast GABA_A_ synapses because the synchronization window is broader.

### The GABA_A_/GABA_B_ Ratio Regulates Synchrony

**Figure 5:**
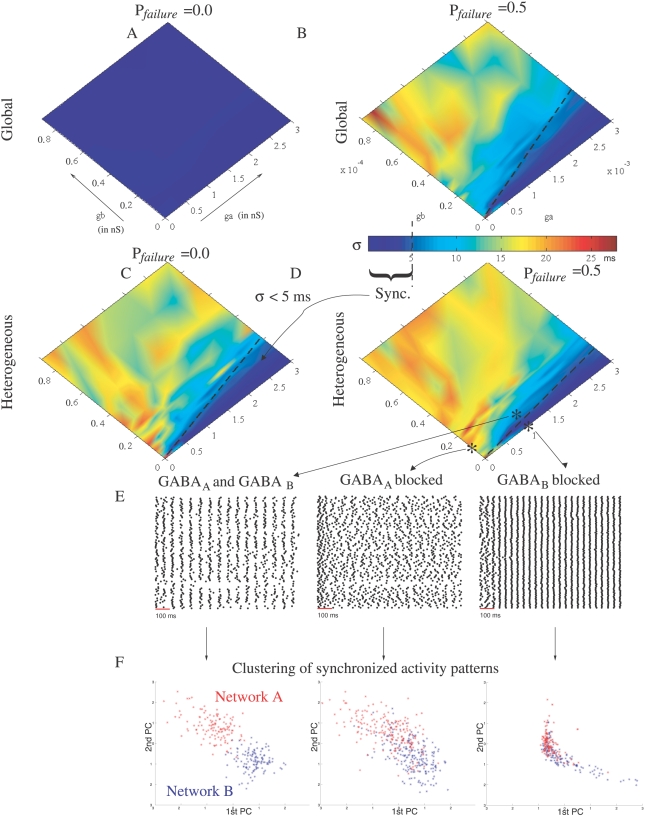
Phase diagrams in the presence of network heterogeneity and/or synaptic failure. (A–D) The synchronous stationary state (sync.) corresponding to *σ*<5 ms is depicted as the blue region. *g_a_* and *g_b_* are expressed in nS and denote the values of the peak conductance *g* in Equation 12 for GABA_A_ and GABA_B_, respectively. The dashed lines separating the synchronous state to the asychronous state were obtained by fitting the contour plot *σ* = 5 ms. The equations of the separating line are *g_a_* = 11*g_b_* (global, *P*_failure_ = 0.5, (B)), *g_a_* = 14*g_b_* (heterogeneous, *P*_failure_ = 0.0, (C) and *g_a_* = 25*g_b_* (heterogeneous, *P*_failure_ = 0.5, (D)). (E) spike rasterplots are indicated for a network (heterogeneous connectivity and *P*_failure_ = 0.5) with intact connections (*g_a_* = 1 nS and *g_b_* = 0.1 nS) and with GABA_A_ or GABA_B_ blocked. (F) Clustering of synchronized activity patterns. Two networks (A and B) of *N* = 100 neurons have been randomly generated with 0.5 probability of connection. At each oscillatory cycle, the network activity is represented as a binary vector in a multidimensional space (*N* = 100), where each dimension corresponds to the binary state of a given PN (1 if synchronized and 0 otherwise). The resolution at which synchronized neurons are determined is *ε* = 5 ms (see Methods). We pooled the binary data obtained at the different oscillatory cycles (extracted between 300 to 3000 ms), for the different networks (A and B) and from repeated trials (3 runs for each network). The data were projected, using logistic PCA [36], onto the first two principal components (PC). Red and blue points in the PCA plane are the projected data for networks A and B, respectively. Left is for intact networks, with GABA_A_ and GABA_B_ coupling (*g_a_* = 1 nS and *g_b_* = 0.1 nS). Middle and right are for GABA_A_ or GABA_B_ blocked, respectively.

In the previous sections, the effect of GABA_A_ or GABA_B_ on synchrony has been studied in isolation. We now consider a network of *N* = 100 neurons coupled with both fast and slow inhibition. A probability of synaptic failure (*P*_failure_ = 0.5 and 0.0) is considered and two patterns of connectivity are taken into account: global (neurons are connected all-to-all) and heterogeneous (neurons are randomly connected with 0.5 probability). Figure 5 presents the spike time jitter estimated from simulations for different values of the GABA_A_ and GABA_B_ conductances *g_a_* and *g_b_*. In the absence of synaptic failure and network heterogeneity, the synchronized state (defined as *σ*<5 ms, blue region in Figure 5) extends to the entire phase space (Figure 5A). In the presence of network heterogeneity and/or synaptic failure, however, the synchronized state depends on the relative amount of received fast and slow inhibition. The dashed lines demarcating the synchronous state are similar in the case of global connectivity and *P*_failure_ = 0.5 (Figure 5B) as well as in the case of heterogeneous connectivity and *P*_failure_ = 0.0 (Figure 5C). Thus, network heterogeneity and synaptic failure play the same role in breaking synchrony. With heterogeneous connectivity and synaptic noise (*P*_failure_ = 0.5), the line demarcating the synchronous state in Figure 5D is *g_a_*/*g_b_*≈25 (*σ*<5 ms when *g_a_*/*g_b_*>25).

In heterogenous networks, the number of GABA_A_ and GABA_B_ inputs differs from one cell to another and thus some neurons exhibit synchronized activity while others do not. If neural assemblies do play a role in sensory representation, then the identities of the synchronized neurons would be reproducible across repeated trials and would be altered by changing the pattern of connections. To test this hypothesis, we performed repeated simulations with two different networks (A and B). Figure 5E shows spike rasterplots obtained from network A with intact connections, and GABA_A_ or GABA_B_ blocked. The state of a PN at each oscillatory cycle is represented as a bit 1 or 0 depending on whether its firing is synchronized or not. At each oscillatory cycle, the stimulus is thus characterized as a point in a multidimensional space, where each dimension corresponds to the binary state of a given PN. Figure 5F shows a 2D projection of these data points. Note that logistic principal component analysis (PCA) has been used for this analysis because it is better suited to modelling binary data than conventional PCA [36]. Two clusters corresponding to networks A and B are well identified with GABA_A_ and GABA_B_ inhibition. These two clusters are almost linearly separable. They overlap, however, when GABA_A_ or GABA_B_ is blocked. These observations indicate that GABA_A_ and GABA_B_ are both needed to create specific assemblies of synchronized neurons.

### Storing Stimulus Patterns in Inhibitory Sub-circuits

**Figure 6:**
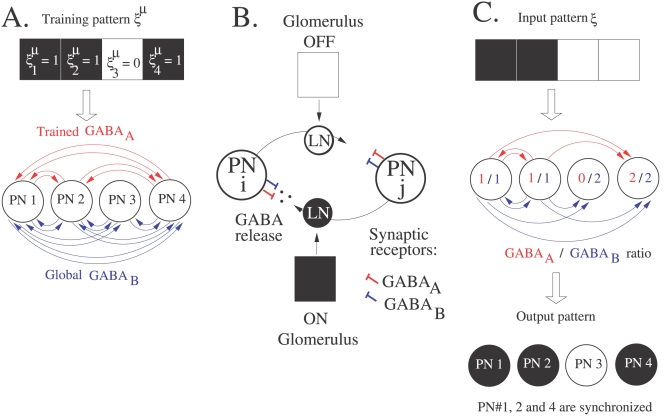
Storage and recall in inhibitory sub-circuits. (A) Trained GABA_A_ connectivity. The spiking associative memory consists of oscillatory PNs (one PN per input component) coupled with GABA_A_ and GABA_B_ synapses. Following clipped Hebbian learning (Equation 7), GABA_A_ connections are created between the first, second and fourth PNs (neurons associated to active bits in the training pattern *ξ^μ^*). For simplicity, we consider that the GABA_B_ network is global. (B) Hypothetical input-dependent gating of lateral inhibition in the AL. Two PNs (PN *i* and *j*) are represented as large circles. Lateral inhibition between PNs is gated by inhibitory LNs (small circles) receiving glomerular input. In the presence of an odor, the active glomerulus (black square) turns on the LN (black circle) associated to the connection *j* → *i*. The LN releases GABA that binds to GABA_A_ and GABA_B_ receptors onto the postsynaptic cell (PN *i*). On the contrary, the inactive glomerulus (white square) turns off the LN (white circle) thereby keeping silent the connection *i* → *j*. (C) Input-dependent gating of lateral inhibition in the spiking associative memory. The input pattern *ξ* (noisy version of the training pattern) activates a specific inhibitory circuit in the GABAergic network depicted in (A). The first and second PNs are associated to active bits in the input pattern *ξ* and their outgoing connections are thus activated. On the contrary, the third PN is associated to an inactive bit in the input pattern and its outgoing connections are turned off. PNs synchronize according to the balance between their GABA_A_ and GABA_B_ inputs (GABA_A_/GABA_B_ ratio). Here, the first, second and fourth PNs synchronize (GABA_A_/GABA_B_≥1) whereas the third PN desynchronizes (GABA_A_/GABA_B_<1) and the training pattern is retrieved (synchronized PNs are black).

In the previous sections, we have shown that synchronized neural assemblies are triggered by GABA_A_ and GABA_B_ connectivity. In the AL of the honeybee, the GABAergic network is functionnally organized to reflect correlations between glomeruli [37]. In *Drosophila*, inhibitory LNs present specificity in their odor responses [14], and this specificity results from repeated exposure to an odor [38]. Therefore, it seems plausible that the GABAergic network exhibits some form of Hebbian synaptic plasticity to store odor stimuli (e.g. [39]). To investigate the problem of learning in inhibitory networks, we use our model to store and recall representations of different input patterns. To store *M* binary patterns *ξ_i_^μ^* ∈ {0,1}(*μ* = 1···*M*, i = 1···*N*), we consider, for simplicity, that the GABA_B_ network is global and that the GABA_A_ network is trained using clipped Hebbian learning :
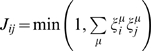
(7)where *J_ij_* = 1 if presynaptic neuron *j* is connected to postsynaptic neuron *i* with a fast GABA_A_ type synapse and *J_ij_* = 0 otherwise. Figure 6A provides an example of GABA_A_ connectivity trained from a single pattern. The PNs in the antennal lobe do not inhibit each other directly but they do so via local neurons. Inhibitory LNs receive direct synaptic input from olfactory receptors [40] and show specificities in their response to odors [14],[38]. Consequently, only a sub-network of the trained connectivity may be activated by the olfactory stimulus. Figure 6B depicts a hypothetical input-dependent gating of lateral inhibition between PNs. To develop this idea further, a GABA_A_ connection in our model is functionally active between neurons *j* and *i* when both *J_ij_* = 1 (connection set by Equation 7) and *ξ_j_* = 1 (reflecting the fact that a putative LN associated with this connection is activated by input *ξ_j_*). A GABA_B_ connection is functionally active between neurons *j* and *i* only when *ξ_j_* = 1 (GABA_B_ connectivity is global in the assumptions derived from our model). Figure 6C depicts the sub-network of GABA_A_ and GABA_B_ connections activated by input pattern *ξ* (noisy version of training pattern *ξ^μ^*). As seen previously, the relative number of GABA_A_ and GABA_B_ inputs modulate the degree of synchrony. In Figure 6C, the third PN desynchronizes because it only receives GABA_B_ inhibition whereas the other PNs synchronize. If state 1 or 0 is assigned to synchronized or desynchronized neurons respectively, then the original training pattern *ξ^μ^* is retrieved.

**Figure 7:**
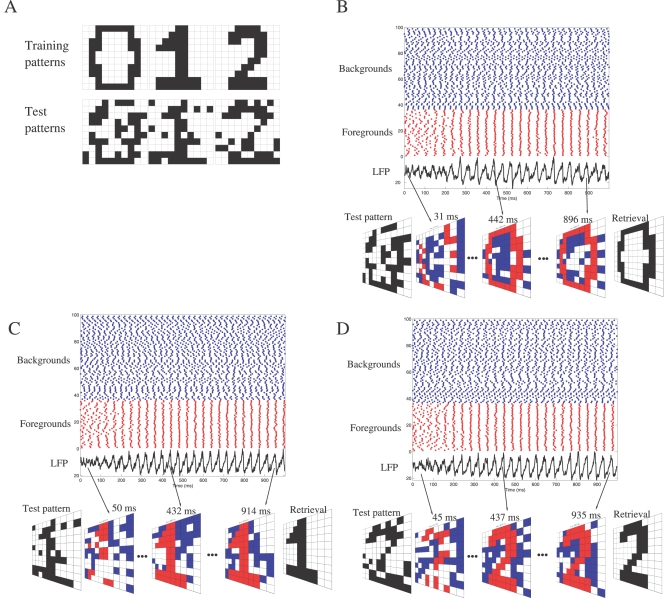
Illustrative example of pattern retrieval. (A) The learning rule Equation 7 is used to train the GABAergic network (N = 100) with the three images ‘0’, ‘1’ and ‘2’, each one having 36 black and 64 white pixels. Test patterns are noisy versions of the training patterns (20% of the pixels are randomly flipped). (B) The noisy version of ‘0’, presented as input, activates a specific sub-circuit of the trained connectivity. The corresponding network is simulated for 1 sec of biological time. Peak conductances *g_a_* = 1 nS and *g_b_* = 0.04 nS have been adjusted according to *g_a_*/*g_b_* = 25 (demarcating the synchronous state in Figure 5D) so that a neuron is synchronized when the number of its GABA_A_ synaptic inputs exceeds that of its GABA_B_ inputs, and is desynchronized otherwise. Neurons that correspond to active and inactive bits in the original training pattern are classified as foregrounds and backgrounds, respectively. In the rasterplot, foreground neurons are artificially grouped to visualize their synchronization (spikes as red dots). Background neurons are desynchronized (spikes as blue dots). The LFP, computed as the average of the PNs' membrane potentials, oscillates at ∼25 Hz. At each cycle, particular neurons fire within a temporal window of ±5 ms around the peak of the LFP. This phase-locked activity is visualized at each LFP cyle (see Video S1 for its evolution). The binary retrieval is formed by assigning bit 1 or 0 to synchronized or desynchronized neurons, respectively. (C) Conventions are similar to (B), except that the noisy version of ‘1’ is presented as input (see Video S2 for the evolution of the phase-locked activity). (D) Conventions are similar to (B), except that the noisy version of ‘2’ is presented as input (see Video S3 for the evolution of the phase-locked activity).

To illustrate the functioning of the spiking associative memory, we used the learning rule (7) to train the GABAergic network with the three black-and-white images ‘0’, ‘1’ and ‘2’ depicted in Figure 7A. Noisy versions of the training patterns, where 20% of the pixels are randomly flipped, are presented as test patterns. Each test pattern activates a sub-circuit of the trained connectivity and the corresponding network is simulated for 1 sec of biological time. Neurons that correspond to active and inactive bits in the original training pattern are classified as foregrounds and backgrounds, respectively. The LFP, computed as the average of the PNs' membrane potentials, oscillates at ∼25 Hz. At each cycle, particular neurons fire within a temporal window of ±5 ms around the peak of the LFP. This phase-locked activity is visualized at each LFP cyle in Figure 7B–D (see also Videos S1, S2, and S3). We observe that foreground neurons synchronize their activity (activity of foreground neurons in red color for both figures and videos), and fire consistently in phase with the LFP at each oscillatory cyle. These foreground neurons form a stable synchronized neural assembly that does not evolve in time. In contrast, background neurons are desynchronized (activity in blue) and fire more or less randomly. If state 1 or 0 is assigned to synchronized or desynchronized neurons respectively, then the retrieval is perfect for the three patterns.

### Storage Capacity Is Similar to that of Willshaw's Network

An important metric of spiking associative memories is capacity. In other words, how many patterns can be stored and retrieved reliably by considering phase-locked neurons? We present a simple analysis that leads to an estimate of the capacity for a network of *N* neurons and provide computer simulations confirming our estimate. In the simulations of the spiking associative memory, the peak conductance values for GABA_A_ and GABA_B_ have been adjusted according to *g_a_*/*g_b_* = 25 (demarcating the synchronous state in Figure 5D) so that a neuron is synchronized when the number of its GABA_A_ synaptic inputs exceeds that of its GABA_B_ inputs and is desynchronized otherwise. The final state of neuron *i* can therefore be written as
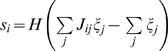
(8)where *H* is the heaviside function and *s_i_* = 1 when neuron *i* is synchronized and 0 otherwise. The binary model defined by Equations 7 and 8 is formally equivalent to Willshaw's model of associative memory [41]. Interestingly, GABA_B_ connectivity plays the same role as the activity dependent threshold in Willshaw's model. A relatively simple analysis of the storage capacity is possible when the input patterns consist of exactly *fN* active bits, where *f* is the input activity. The storage capacity *α*_c_ (in terms of maximum number of patterns per neuron) obtained analytically in [42] for the Willshaw's model is
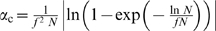
(9)


**Figure 8:**
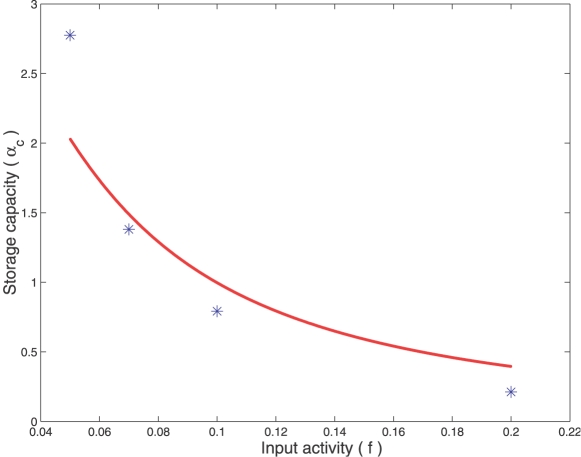
Estimation of the storage capacity. The storage capacity (*α*_c_) is expressed in terms of maximum number of patterns stored per neurons. It is plotted as a function of the input activity (*f*), i.e. the input patterns consist of exactly *fN* active bits. The size of the network is *N* = 100. The plain curve is the theoretical storage capacity derived for Willshaw's model (Equation 9). Stars represent the storage capacity estimated for the spiking neural network working as a phase-locked associative memory (see Methods). For *f* = 0.1 and 0.2, the GABA_A_ and GABA_B_ peak conductances are *g_a_* = 0.25 nS and *g_b_* = 0.01 nS. For *f* = 0.05 and 0.07, *g_a_* = 0.5 nS and *g_b_* = 0.02 nS.

Figure 8 compares the storage capacity *α*_c_ given by Equation 9 to the one estimated numerically for our spiking associative memory (see Methods). As seen in the figure, the spiking network possesses storage capacities similar to those of conventional associative memories such as the Willshaw's model. The storage capacity is optimal in the sparse coding regime, where *f*≈ln *N*/*N*. Above this threshold, performance drops significantly.

## Discussion

PN synchrony has been observed in the AL of the locust [4],[5], of the bee [6] and the moth [7],[8]. In *Drosophila*, PNs are inhibited via at least two distinct conductances, GABA_A_ and GABA_B_ [14]. GABA_B_ postsynaptic potentials present a much slower decay rate than the ones produced by GABA_A_ inhibition. By means of computational modelling, we investigated the roles of fast and slow inhibition in spike timing precision and neuronal synchrony.

### Opposite Roles of Fast and Slow Inhibition

We first mimicked somatic injection of hyperpolarizing current into individual cells. Our simulations show that the spike time jitter decreases with the duration of the injected current pulse (Figure 1). This observation is in agreement with in vitro experimental recordings [28], because the hyperpolarizing current pulse, injected into the cells, is reproducible across repeated trials. In a network of coupled neurons, however, variable inhibition may come from heterogeneous connectivity or from the presence of synaptic failures, both being likely to occur in vivo. How does this variability affect the spike timing precision in PNs? Computer simulations and analytical results predict that the spike time jitter is proportional to the decay time constant of the inhibitory synapse (Equation 2 and Figure 2). Hence, variable inhibition received on slow GABA_B_ synapses leads to unpredictible firings, whereas variable inhibition is especially tolerated with fast GABA_A_ synapses. Another way to produce long-lasting inhibition is by asynchronous GABA release. We demonstrate that the slow inhibition which results from the summation of many asynchronous synaptic events accentuates temporal dispersion (Equation 4 and Figure 3).

Our model predicts that fast and slow inhibition play opposite roles in PN synchrony; fast inhibition synchronizes whereas slow inhibition desynchronizes (see rasterplots in Figure 5E). Several studies show that PN synchronization is induced by GABA_A_ inhibition [12],[10],[9]. When GABA_A_ inhibition is pharmacologically blocked by local injection of picrotoxin into the AL, PN synchronization and field potential oscillations are lost. Evidence in favour of a desynchronization mechanism by GABA_B_ is provided by in vivo PN recordings : the spike time jitter decreases in PNs in the presence of the GABA_B_ antagonist CGP54626 (see Figure 4 in [14]). Additional indirect confirmation could be obtained by observing whether the oscillatory power of a recorded field potential increases when the GABA_B_ synapses are blocked, which would imply that more PNs are synchronized in the absence of GABA_B_ inhibition. A more direct confirmation would require the indexing of PN firings with respect to the common field potential, and analysis of the phase histogram in the control condition and in the presence of the GABA_B_ antagonist. According to our model's prediction, the PN firing phases should be more broadly distributed in the control condition.

A related study on spike-time reliability was published while this manuscript was under review. In [43], it is shown that fast synaptic fluctuations increase spike timing precision and synchronization, whereas slower input fluctuations have the opposite effects. This finding is in agreement with our results showing that fast, noisy GABA_A_ inputs improve synchrony, whereas, slow, noisy GABA_B_ inputs destroy it. In [43], we note however that the neural response becomes unpredictible for very fast input fluctuations (time scale <2 ms), a behavior neither observed in our simulations nor predicted by our theory. This discrepancy may result from differences in experimental conditions. The study in [43] was concerned with reliability in single neurons driven by aperiodic inputs, whereas in this article, we have focused on synchronization of coupled neurons receiving periodic GABAergic inputs.

### Frequency of Network Oscillations

Little evidence for LFP oscillations has been found in *Drosophila* [14]. It is possible that a coherent population oscillation hardly emerges from a network with a limited number of neurons (only 150 *Drosophila* PNs [44],[45]). In the case where field oscillations are observed, their frequency is less than 4 Hz [18]. This is low in comparison to the 20–30 Hz frequency range encountered in other insect species which include the wasp, locust, cockroach and honeybee [6]. It is known that the decay time constant of the inhibition controls the frequency of the oscillations in inhibitory networks [29],[2],[35]. In agreement with this result, we found in our model that frequency is higher with fast inhibition (F ∼20 Hz with *τ*_GABA_ = 10 ms). The period of the network oscillation increases linearly with *τ*_GABA_ (see Figure S1) so that a 4 Hz frequency (period = 250 ms) is obtained when *τ*_GABA_ = 345 ms. This observation is compatible with the time decay of the CGP54626-sensitive component observed in the *Drosophila* PNs [14]. We therefore predict that the 4 Hz LFP frequency observed in *Drosophila* is mainly due to strong GABA_B_ inhibition which masks the effects of GABA_A_. This prediction could be tested experimentally by observing whether the frequency of the field oscillation reaches the 20–30 Hz frequency range in the presence of a GABA_B_ antagonist.

### Stable Neural Assemblies

Our AL model converges onto assemblies of synchronized neurons triggered by the GABAergic network (Figure 5). The relative number of received GABA_A_ and GABA_B_ inputs regulates synchrony and determines whether particular neurons engage in neural assemblies. These assemblies do not evolve in time (stable synchrony). Our work differs from previous theoretical studies in which the stimuli are encoded by transient synchrony, i.e., the subset of synchronized neurons changes over time [46],[17]. In previous studies, transient synchrony is achieved by temporal variations of the fast GABA_A_ input. The most active LNs inhibit the others and may even suppress their activity due to strong LN-LN inhibition. These active LNs, however, increase their adaptation current, which makes subsequent firing harder. Such a fatigue mechanism leads to a complex time-varying competition between LNs that may depend on which LNs win the competition first. In contrast, the neural assemblies created by our mechanism are stable and do not depend on the initial state of the network, synchronized or not. Although LNs have not been used explicitly in our model, we propose another potential role for inhibitory local neurons (see below). Another difference with [46],[17] concerns the role of slow inhibition: in [17], slow inhibition is introduced to obtain some temporal patterning associated with neural synchrony, whereas, in our study, slow inhibition is introduced to desynchronize PN activity in the presence of synaptic failure.

### Potential Roles for Local Neurons

Modelling early olfactory systems as a network of neurons coupled with inhibition is not uncommon, see for example [47]. In our study, we used a simplified model of the insect AL that allows for analytic calculations. Inhibitory LNs were not considered explicitly in the mathematical derivation of the spike time jitter for the PN population (see Text S1). However, the spike time jitter is not affected when our AL model is complemented with inhibitory local neurons (Figure S3). The inhibitory LNs in the extended model fire in synchrony, despite asynchronous PN activities. A potential role for inhibitory LNs in the antennal lobe is to produce stimulus-specific spatial patterns of inhibition. In the antennal lobe, inhibitory LNs receive direct synaptic input from olfactory receptors [40] and present specificities in their response to odors [14],[38]. Consequently, we hypothesized that lateral inhibition between PNs is mediated by the olfactory stimulus. We proposed an input-dependent gating mechanism of lateral inhibition between PNs so that stimulus patterns trigger specific inhibitory sub-circuits (see Figure 6). As particular neurons synchronize or desynchronize according to the inhibition received, neural assemblies are adjusted by stimulus-induced changes in inhibitory sub-circuits. It has recently been shown that LNs are not only inhibitory. A new class of excitatory cholinergic LNs has been identified in the *Drosophila* AL [48],[49]. We have complemented our AL model with excitatory cholinergic synapses between PNs and show that lateral excitation redistributes activity over the ensemble of PNs so that all neurons fire, even those not receiving an external stimulation (Figure S2). This result is consistent with the observation that excitatory LNs in the AL form a dense network of lateral excitatory connections that may boost weak PNs above the firing threshold [48].

### Storing Stimulus Patterns in Inhibitory Sub-Circuits

To assess whether inhibitory sub-circuits are capable of memory storage, we considered that the GABA_B_ connectivity is fixed and global and that the GABA_A_ connectivity is trained according to the Hebbian axiom “cells that fire together, wire together”. We showed that lateral GABA_A_ connections set by Hebbian learning endow the spiking network with properties of binary associative memories (Figure 6). The activity of the spiking network converges towards fixed point attractors (assemblies of synchronized neurons) determined by the pattern of connectivity (Figure 7 and Videos S1, S2, and S3). Binary vectors are stored and retrieved as synchonized neural assemblies (as corresponding to 1 if a neuron is synchronized and to 0 otherwise). We do not claim that this model is biologically plausible or mathematically optimal, but we claim it accounts for some biological observations and allows a simple analysis of the estimation of storage capacity.

A memory trace of synchronized neural activity compatible with short-term Hebbian plasticity has been revealed in the AL of honeybees [39]. A functionally organized inhibitory network, whose connectivity reflects correlations between glomeruli, best reproduces the experimental data [37]. In *Drosophila*, inhibitory LNs present specificity in their odor responses [14], that results from repeated exposure to an odor [38]. It is therefore plausible that the GABAergic network exhibits some form of Hebbian synaptic plasticity enabling the storage of odor stimuli. Evidence for synaptic plasticity in inhibitory networks, however, is scarse and remains controversial. Very few research has addressed the issue of plasticity at inhibitory synapses in oscillatory networks [50],[51]. Much work in synaptic plasticity has focused on excitatory synapses. Excitatory synapses of PNs onto inhibitory LNs may also be a site for synaptic plasticity. According to our simplified model, an increase of the LN's excitatory conductance would lead to greater GABA release and thereby the “effective” inhibitory connections between PNs would be modified (Figure 6B). Such an increase of inhibitory transmitter release after long-term plasticity at excitatory synapses has been observed in cerebellar stellate cells [52].

The storage capacity of our simplified AL model is comparable to that of classical binary-coded models like Willshaw's network (Figure 8). Good performance in terms of stored patterns per neuron is reached when the activity in the network is sparse (very low fraction of synchronized neurons at each LFP cycle). It would be interesting to see whether odors are sparsely represented by the PN population in the AL, as experimental data about sparseness of PN activity is contradictory in *Drosophila* [53],[54]. To estimate storage capacity, we deliberately considered a simplified model of the AL. The first simplification is to use binary stimulus patterns. Considering binary glomerular response (active or inactive) is not uncommon, e.g., [55],[56]. In the case of insects, however, it may be too restrictive. The dose-response curves for honeybees' glomeruli is well described by a smooth sigmoid function with estimated Hill slope parameters in the range 0.14–0.56 [57]. Therefore, further work is necessary to take into account graded glomerular responses in our model. The second simplification is the use of a global GABA_B_ network. Actually, the odor-evoked GABA_B_ inhibition in *Drosophila* has been shown to differ across glomeruli and odors [14]. Training both GABA_A_ and GABA_B_ connections would have the merit to convey complementary pieces of information. Fast and slow inhibition could therefore multiplex information into separate channels, in agreement with recent experimental work [11].

## Methods

### Neuron Model

PNs are modelled as quadratic integrate-and-fire (QIF) neurons [58]. The evolution of the membrane potential *V* is described by:
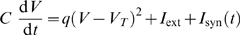
(10)where *I*_syn_(*t*) is the received synaptic current and *I*_ext_ = *I*+*I*_inj_−*I*_th_ is a constant external current. *I* represents a driving current, *I*_inj_ is an injected current, and *I*_th_ denotes the rheobase, i.e., the minimal current required for repetitive firing. The QIF neuron fires as soon as *V* reaches the threshold *V*_th_. Right after the spike, *V* is reset to the value *V*_reset_.

In the absence of synaptic current, the QIF neuron presents two distinct regimes depending on the sign of the external current. When *I*_ext_<0 there are two fixed points. The stable ones defines the resting potential
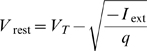
(11)


**Figure 9:**
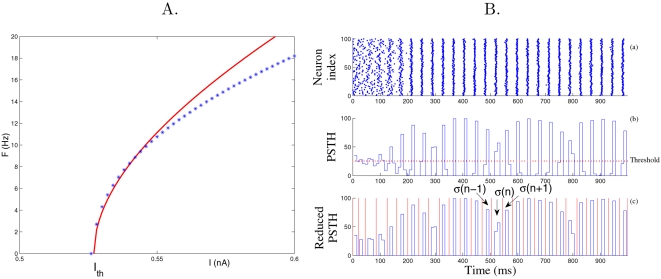
Frequency-current response curve of the PN model (A): Firing rate *F* versus applied current *I*, and estimation of the spike time jitter (B). (A) The curve is for our PN model (Equation 10 with *I*_inj_ = 0). Stars are for the simulations of the conductance-based PN model from [17],[46]. As expected, our PN model is a good approximation of the type 1 conductance-based model around the rheobase *I*_th_ [58]. (B) The rasterplots (a) of PNs are integrated over time bins of 5 ms yielding a peri-stimulus histogram (PSTH, see b). The PSTH is further reduced by cutting above the threshold (dotted line in b) corresponding to the mean firing rate (yielding reduced PSTH, see c). From the reduced PSTH, consecutive slots of activity (in red in c) are extracted and the spike time jitter *σ*(*n*) is computed as the standard deviation of the spike times falling into each slot *n*. The spike time jitter at convergence *σ* is the one obtained at the end of the simulation.

The unstable one is the threshold above which the neuron fires a single spike. When *I*_ext_>0 the QIF neuron fires regularly and the firing frequency scales as 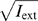, as in type 1 neurons. The QIF model represents the normal form of any type 1 neurons near the saddle-node bifurcation and is related to the so-called *θ*-neuron [58]. Since the QIF neuron is expected to reproduce the characteristics of any type 1 neuron close to bifurcation, it has been widely used as a realistic neuron model [26]. Parameters in Equation 10 were chosen as to obtain a frequency-current response similar to the PN conductance based model by [17],[46] (see Figure 9A): *C* = 0.143 nF, *V_T_* = −41.18 mV, *q* = 9.29×10^−4^ mS V^−1^, *I*_th_ = 0.527 nA, *V*_th_ = 30 mV and *V*_reset_ = −70 mV. From Equation 11, *V*_rest_ = −65 mV when *I*_ext_ = −*I*_th_.

### Synaptic Current Model

The synaptic current *I*_syn_ in Equation 10 results from the integration of GABAergic currents over the dendritic tree. At each synapse, *I_GABA_* (in nA) is given by
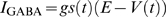
(12)where *E* is the reversal potential of the synapse (*E* = −70 mV for GABA_A_ and −95 mV for GABA_B_) and *g* is the peak synaptic conductance in µS. The GABA_A_ peak conductance *g_a_* is in the range (0.25–1.2)×10^−3^ µS and the GABA_B_ peak conductance *g_b_* is in the order of 0.06×10^−3^ µS [59]. Conductance kinetics are modelled by decaying exponentials
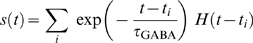
(13)where the *t_i_* are the times of the synaptic events and *τ*_GABA_ is the synaptic time decay (*τ*_GABA_ = 10 ms for GABA_A_ and 100 ms for GABA_B_). The Heaviside function *H* ensures causality.

Inhibitory interneurons may release transmitters synchronously or asynchronously [30],[31]. When synchronous release is considered, the times of the synaptic events are given by *t_i_* = *t_i_^f^*+Δ where the *t_i_^f^* are the firing times of the presynaptic neuron and Δ = 5 ms is the propagation delay. When asynchronous release is considered, each pre-synaptic spike triggers a number of GABAergic post-synaptic events. These events are triggered asynchronously, according to an exponential distribution of standard deviation *λ*. The probability that a presynaptic spike at time *t_i_^f^* produces a post-synaptic event at time *t_i_* is described by:
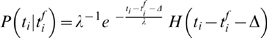
(14)


### Network Model

Not all the PNs fire in the presence of an odor. In the Locust for example, about 100 PNs (out of 830) are activated by the presentation of an odor [60]. The network used in the simulation is a matrix of *N* = 10×10 neurons corresponding to these odor-responding PNs. We take *I* = 0.75 nA in Equation 10 so that, without synaptic coupling, PNs are oscillators firing at the same frequency (about 40 Hz). In the network, PNs are coupled directly via GABAergic synapses. Inhibitory LNs are not modelled explicitly because of the lack of experimental data concerning the functionning of LNs and because of LN diversity. We consider two types of inhibitory synapses, GABA_A_ and GABA_B_, and a probability of synaptic failure (unless specified otherwise, *P*_failure_ = 0.5). The network was programmed in C and simulated with a fourth-order Runge-Kutta integration method with a time step of 50 µs. The initial network condition corresponds to a completely desynchronized neuronal population. This is obtained from the following procedure. The firing times *T* of the neurons are given by integrating Equation 10 with *I*_syn_ = 0 from their initial membrane potentials *V*(0) to the firing threshold *V*(*I*) = *V*_th_

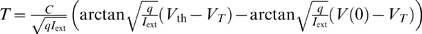



The maximum firing time *T*_max_ is obtained when *V*(0) = *V*_reset_. This firing time equation is then solved for *V*(0)
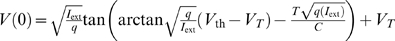



The above equation provides initial membrane potentials *V*(0) for firing times *T* taken randomly between 0 and *T*_max_. This initialization procedure of the PNs ensures firing times uniformly distributed over (0, *T*_max_).

### Data Analysis

The estimation procedure for the spike time jitter *σ* is similar to the one in [61] and is described in the caption of Figure 9B.

Estimation of the phase-locking probability closely matches the protocol in Figure 9B to determine slots of activity. In each slot, the mean firing time *T̅* of the neuronal population is computed and the phase-locking probability is obtained by counting the relative number of spikes falling into a bin of ±*ε* ms around the mean firing time *T̅* (*ε* = 5 ms for data in Figure 4C and *ε* = 1 ms for data in Figure 4D).

The critical storage capacity *α*_c_ is defined as the maximum number of patterns per neurons that can be stored and retrieved reliably. For the numerical estimation of *α*_c_, binary patterns with *fN* active bits are stored using the clipped Hebbian learning rule on GABA_A_ synapses (Equation 7). Each individual pattern however elicits a specific sub-network of GABA_A_ and GABA_B_ coupling (as described in the Results section). For each pattern, its corresponding sub-network is simulated for 3 s of biological time, starting from a completely desynchronized state. Consecutive slots of activity are determined as in Figure 9B. The spike time jitter *σ* is computed for each neuron as the standard deviation of its firing times over the last activity cycles. To form a binary output, *fN* phase-locked neurons (with the smallest *σ*) are considered as active bits and the remaining (1−*f*)*N* neurons (with higher *σ*) are inactive bits. All the stored patterns are considered to be retrieved reliably if the mean overlap between stored and retrieved patterns exceeds 0.9. The above procedure is repeated with a larger number of stored patterns until the patterns can no longer be retrieved reliably. Each storage capacity estimated in Figure 8 has been obtained by averaging the results over five runs.

## Supporting Information

Text S1 Spike time jitter of the PN population.
(0.04 MB PDF)

Figure S1 The synaptic parameters control the period of the network oscillation. Period of the network oscillation versus parameters of the GABAergic synapses (time constant and synaptic conductance).
(0.02 MB PDF)

Figure S2 AL model with PN-PN excitatory connections.
(0.03 MB PDF)

Figure S3 AL model with inhibitory LNs.
(0.38 MB PDF)

Video S1 Phase-locked activity of the spiking associative memory for noisy ‘0’ pattern. The neurons which correspond to black and white pixels in the original training pattern are classified as foregrounds and backgrounds, respectively. The network is simulated for 1 sec of biological time. The LFP, computed as the average of the PNs' membrane potentials,oscillate at ≍ 25 Hz. At each cycle, foreground and background neurons, firing within a temporal window of ? ms around the peak of the LFP, are shown as red and blue pixels, respectively.
(8.30 MB AVI)

Video S2 Phase-locked activity of the spiking associative memory for noisy ‘1’ pattern. Conventions are similar to Video S1
(8.30 MB AVI)

Video S3 Phase-locked activity of the spiking associative memory for noisy ‘2’ pattern. Conventions are similar to Video S1.
(8.30 MB AVI)
