# Supplementary material for: A Model of Stimulus-Specific Neural Assemblies in the Insect Antennal Lobe
Source: PLoS Comput Biol. 2008 Aug 1;4(8):e1000139. doi: 10.1371/journal.pcbi.1000139 (PMC2536510; doi:10.1371/journal.pcbi.1000139)
Supplement: Text S1 — Spike time jitter of the PN population. (0.04 MB PDF) [file pcbi.1000139.s001.pdf]

Spike time jitter of the PN population

The PN population is modelled as a set of QIF neurons coupled with mutual inhibition (see Methods). A QIF neuron that is strongly inhibited cannot fire until the inhibition has worn off enough. Whatever the initial condition might be, the synaptic current is approximately equal to $I^\star$ at the firing time (see phase plane analysis in [1]). The firing time is thus relatively independent of the initial condition $V(t=0)$ [1, 2]. We first consider a PN receiving a burst of k inhibitory synaptic events at times $t_1, t_2 \cdots t_k$ so that the synaptic current at the firing time $T > t_k$ is

$$I^\star \approx I_{syn}(T) = \sum_{i=1}^{k} g e^{-(T-t_i)/\tau_{GABA}} (E_{GABA} - V_{th})$$

and thus,

$$T \approx \tau_{GABA} \left(\ln g - \ln(I^\star) + \ln(E_{GABA} - V_{th}) + \ln \sum_{i=1}^{k} e^{t_i/\tau_{GABA}} \right) \tag{A-1}$$

Due to the presence of synaptic failures, the number of received synaptic events as well as their timing is variable across neurons and repeated trials. How does this variability affect spike timing precision? To answer this question, we consider as in [3] that the number k of received synaptic events is a random variable with mean $<\!k\!>$ and variance σ_k^2, and the times of the synaptic events t_i are drawn randomly from an unknown probability density function with variance σ_t^2. The only random variable in Eq. (A-1) is

$$X = \tau_{GABA} \ln \sum_{i=1}^{k} e^{t_i/\tau_{GABA}}$$

Furthermore, we have $\sigma_T^2 = \sigma_X^2$. An approximation for σ_T^2 can be found by considering the fact that the variance of a sum of a random number k of independent random variables x_i, each with variance σ_x^2, is $<\!k\!>\ \sigma_x^2 +\ <\!k\!>^2\ \sigma_k^2$, and that the variance of a function $y = g(x)$ of a random variable x approximately depends on the mean η_x and variance σ_x^2 of x : $\sigma_y^2 \approx |dg/dx|^2_{x=\eta} \sigma_x^2$ (eq. 5-56 in [4]). Using these formulae, we found in [3]

$$\sigma_T^2 \approx \frac{\sigma_t^2}{<\!k\!>} + \tau_{GABA}^2 \frac{\sigma_k^2}{<\!k\!>^2} \tag{A-2}$$

where σ_k^2 and σ_t^2 is the variance in the number and timing of the synaptic events received within the burst of inhibition, respectively.

To derive the spike time jitter of the PN population, we now replace the burst of inhibition with all-to-all inhibitory coupling between N neurons and consider unreliable synapses ($P_{failure}$ =probability of synaptic failure). The spike time jitter of the PN population at the n-th cycle is $\sigma^2(n) = \sigma_T^2$. The inhibitory burst is triggered by the activity of the PNs at the previous cycle $n-1$. The variance in the number of the synaptic events is $\sigma_k^2 = N P_{failure}(1 - P_{failure})$. When the release of GABA is synchronous with the arrivals of the presynaptic spikes, the variance in the timing of the synaptic events is $\sigma_t^2 = \sigma^2(n-1)$. Replacing these expressions in Eq. A-2 leads to

$$\sigma^2(n) \approx \frac{\sigma^2(n-1)}{<\!k\!>} + \tau_{GABA}^2 \frac{\sigma_k^2}{<\!k\!>^2} \tag{A-3}$$

When GABA is released asynchronously, a synaptic event may be released at a time $t_i = t_i^f + \delta t$ well after the arrival of an action potential on a synapse. The time t_i^f of the presynaptic spike is a random variable with variance $\sigma^2(n-1)$. The delay $\delta t = (t_i - t_i^f)$ is given by the exponential distribution with variance λ^2 (see Methods). Therefore, $\sigma_t^2 = \sigma^2(n-1) + \lambda^2$. For asynchronous release, the spike time jitter of the PN population is therefore

$$\sigma^2(n) \approx \frac{\sigma^2(n-1) + \lambda^2}{<k>} + \tau_{GABA}^2 \frac{\sigma_k^2}{<k>^2} \tag{A-4}$$

References

[1] C. Börgers and N. Kopell, "Synchronization in networks of excitatory and inhibitory neurons with sparse, random connectivity," *Neural Computation*, vol. 15, pp. 509–538, 2003.

[2] N. Kopell and G. B. Ermentrout, "Chemical and electrical synapses perform complementary roles in the synchronization of interneuronal networks," *PNAS*, vol. 101, pp. 15482–15487, 2004.

[3] D. Martinez, "Oscillatory synchronization requires precise and balanced feedback inhibition in a model of the insect antennal lobe," *Neural Comp.*, vol. 17, pp. 2548–2570, 2005.

[4] A. Papoulis, *Probability, random variables, and stochastic processes, second edition.* McGraw-Hill Book Co., New York, 1984.
