# Supplementary material for: A Model of Stimulus-Specific Neural Assemblies in the Insect Antennal Lobe
Source: PLoS Comput Biol. 2008 Aug 1;4(8):e1000139. doi: 10.1371/journal.pcbi.1000139 (PMC2536510; doi:10.1371/journal.pcbi.1000139)
Supplement: Figure S1 — The synaptic parameters control the period of the network oscillation. Period of the network oscillation versus parameters of the GABAergic synapses (time constant and synaptic conductance). (0.02 MB PDF) [file pcbi.1000139.s002.pdf]

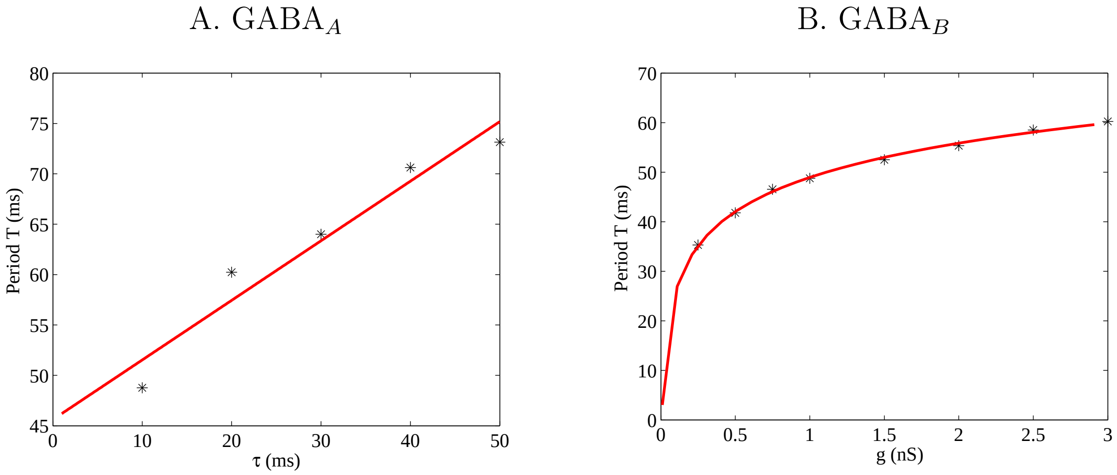

Figure S1: **The synaptic parameters control the period of the network oscillation.** We simulated a network of $N = 100$ neurons (PN models) coupled all-to-all with inhibition ($P_{failure} = 0.5$). We varied the synaptic time decay τ and the synaptic conductance g. The frequency of the network oscillation is given by the frequency of the maximum Fourier component in the power spectrum computed on the average of the PNs' membrane potentials. (A) The period T increases linearly with τ. The stars are simulation data and the solid line is a linear fit. (B) The period T grows as $\ln g$. The stars are simulation data and the solid curve corresponds to the fit $T = a \ln g + b$. The fitted parameter is $a = 9.97$ ms which matches the synaptic time decay $\tau = 10$ ms used in the simulations. Simulation data are in agreement with Eq. A-1 (see Supporting Text S1).
