# Supplementary material for: A Model of Stimulus-Specific Neural Assemblies in the Insect Antennal Lobe
Source: PLoS Comput Biol. 2008 Aug 1;4(8):e1000139. doi: 10.1371/journal.pcbi.1000139 (PMC2536510; doi:10.1371/journal.pcbi.1000139)
Supplement: Figure S2 — AL model with PN-PN excitatory connections. (0.03 MB PDF) [file pcbi.1000139.s003.pdf]

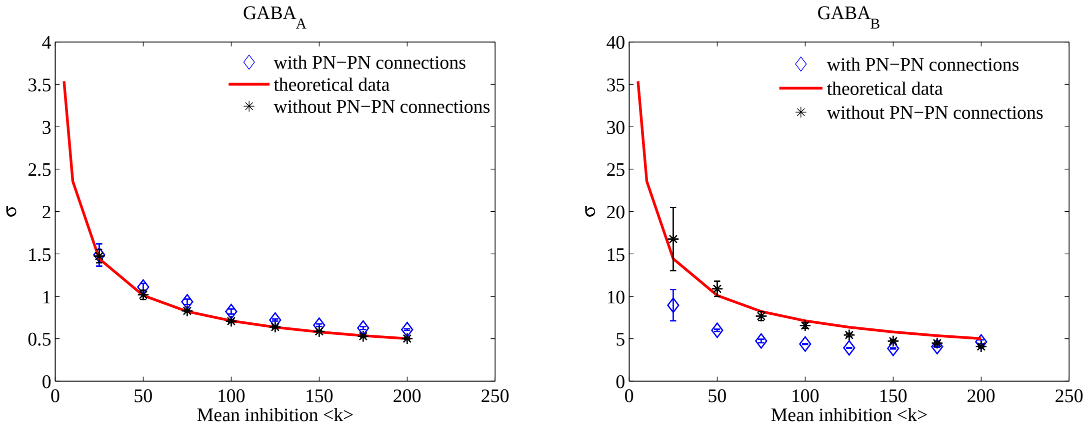

Figure S2: **AL model with PN-PN excitatory connections**. We complemented the AL model ($P_{failure} = 0.5$, all-to-all inhibitory coupling) with excitatory cholinergic synapses between PNs (peak conductance $g_{nACh} = 1nS$, reverse potential $E_{nACh} = 0mV$, synaptic decay $\tau_{nACh} = 10ms$). For most of the PNs (90 out of 100), we take $I = 0$ so that the external current is negative ($I_{ext} = -I_{th}$ in PN equation, see Methods). The excitatory synaptic current, however, is sufficient to boost the weak PNs above the firing threshold. The role of lateral excitation is therefore to spread activity over the entire network so that all neurons fire, even those not receiving an external stimulation. To test the effect of combined lateral excitation and inhibition on PN synchrony, we repeated the simulations of Fig. 2 (G) with the extended AL model. Left and right plots show the spike time jitter (σ in ms) versus the mean inhibition ($<k>= N(1 - P_{failure})$) for GABA$_A$ ($g_a = 2nS$) and GABA$_B$ ($g_b = 0.3nS$) coupling, respectively. The solid curves are theoretical values provided by Eq. 2. Blue diamond-marks correspond to the spike time jitter obtained for the extended AL model (lateral excitation combined with inhibition). Means and standard deviations are computed over five runs. The present data need to be compared to those obtained without PN-PN excitation (black stars). We observe a perfect match when lateral excitation is combined with GABA$_A$ (left plot). Eq. 2 still holds for the extended AL model. The spike time jitter obtained when lateral excitation is associated to GABA$_B$ is lower than the one obtained without lateral excitation (right plot). This additional synchronization is induced by lateral excitation.
