# Supplementary material for: A Model of Stimulus-Specific Neural Assemblies in the Insect Antennal Lobe
Source: PLoS Comput Biol. 2008 Aug 1;4(8):e1000139. doi: 10.1371/journal.pcbi.1000139 (PMC2536510; doi:10.1371/journal.pcbi.1000139)
Supplement: Figure S3 — AL model with inhibitory LNs. (0.38 MB PDF) [file pcbi.1000139.s004.pdf]

Figure S3: **AL model with inhibitory LNs.** We complemented the AL model with inhibitory local neurons. LNs were modelled like the PNs, except that $I_{th} = 0.8$ (see Method). The external current I_{ext} is negative for the LNs so that additional excitatory synaptic current coming from the PNs is needed to generate LN spikes. The network consists of the following connections: cholinergic excitatory synapses from PNs to LNs, GABA_A inhibitory synapses between LNs and GABA_A or GABA_B inhibitory synapses from LNs to PNs. The failure probability for all the synapses is $P_{failure}$=0.5. We varied the number of LNs from $N_{LN} = 30$ to 400 while keeping the number of PNs fixed at $N_{PN} = 100$. Panels from A to D show the rasterplots with GABA_A or GABA_B coupling from LNs to PNs and with $N_{LN} = 30$ or 80. We observe that GABA_A inhibition synchronizes PNs (panels A and C) whereas GABA_B inhibition desynchronizes PNs (panels B and D). Panels E and F show the spike time jitter (σ in ms) versus the mean inhibition ($<k>= N_{LN}(1 - P_{failure})$) with GABA_A and GABA_B coupling, respectively. Plain curves are theoretical values provided by Eq. 2. Blue diamond-marks correspond to the spike time jitter obtained for the extended AL model with inhibitory LNs (means and standard deviations computed over five runs). These data need to be compared to those obtained without LN (black stars). We observe that Eq. 2 holds for the extended AL model. The data obtained with GABA_A coupling from LNs to PNs perfectly matches those obtained without LN (panel E). With GABA_B coupling however, the spike time jitter obtained without LN is slightly above the one obtained with LNs (panel F). When PNs inhibit each other directly via GABA_B synapses, additional jitter comes from desynchronized inhibitory synaptic events. In the network with LNs, lateral GABA_A synapses between LNs lead to synchronized bursts of inhibition even in the presence of desynchronized PNs (see panels B and D).

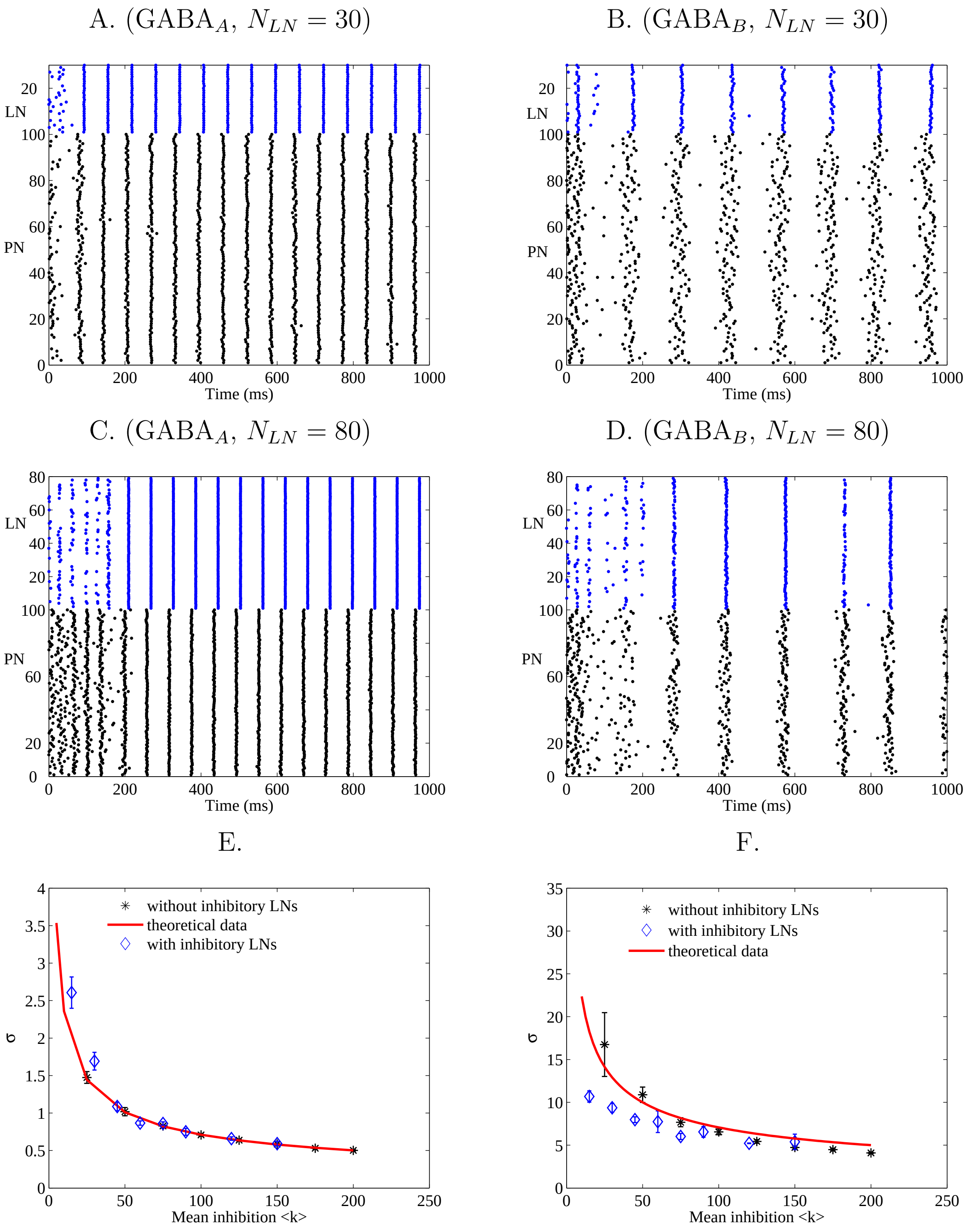

Figure S3: See previous page for figure caption
